# Rupture of an accessory spleen caused by blunt trauma

**DOI:** 10.1007/s00068-024-02591-y

**Published:** 2024-07-09

**Authors:** Agata Grochowska, Piotr Arkuszewski

**Affiliations:** 1 Department of Biomedicine and Experimental Surgery, Student Scientific Club of Biomedicine and Experimental Surgery, Medical University of Lodz, 60 Narutowicza Street, Lodz, 90-136 Poland; 2 Department of Biomedicine and Experimental Surgery, Medical University of Lodz, 60 Narutowicza Street, Lodz, 90-136 Poland

**Keywords:** Blunt trauma, Accessory spleen, Laparotomy, Splenosis

## Abstract

**Purpose:**

The accessory spleen is quite a common abdominal anomaly. However, the traumatic accessory spleen rupture is an extremely rare condition requiring surgical intervention, even laparotomy. 9 cases of traumatic accessory spleen were found published between 1962 and 2022. The study aims to evaluate traumatic accessory spleen rupture cases regarding their causes, clinical course, and possible diagnosis without surgery and treatment.

**Methods:**

Desk research method using available online databases. Descriptive methods were employed to analyze the collected data. The results are summarized in the Table concerning gender, age, injury details, accessory spleen injury characteristics, treatment, and others such as previous splenectomy or primary spleen involvement in injury or accompanying abdominal injuries.

**Results:**

In total, there were 9 cases of traumatic accessory spleen, of which 2 were managed conservatively and the remaining 7 were treated operatively. All the patients survived. One-third of all included patients already had their primary spleen removed, which facilitated the diagnosis of traumatic rupture of an accessory spleen. The proper diagnosis of an accessory spleen rupture was concluded in 2 cases and confirmed in surgery.

**Conclusion:**

The recognition of the traumatic rupture of an accessory spleen before surgery is challenging but can be made easier if the patient underwent splenectomy before. The traumatic accessory spleen rupture does not coexist with an injury of a primary spleen.

## Introduction

The accessory spleen (AS) is splenic tissue manifested as a congenital nodule formed separately from the primary spleen [1–3]. Its appearance is a result of the inability of the primordial splenic buds to fuse in the dorsal mesogastrium in the fifth week of embryonic development [4, 5]. Compared to splenosis, an acquired anomaly occurring following rupture of the spleen with its fragmentation or splenectomy, the AS expresses regular splenic tissue features on histopathological examination and is supplied by the branches of the splenic artery [6]. Predominantly, the AS is an atypical body organ that seldom manifests clinical symptoms. However, it can be the cause of recurrent or acute abdominal pain [7–9]. Its recognition is usually incidental during imagining studies such as CT or during surgery, mainly laparotomy. The prevalence is estimated to be over 10% in autopsy studies [10, 11], around 11-18% in CT [12, 13], and around 15% in laparotomies [14, 15], peaking in North America and Europe. Roughly one-fourth of individuals within the population presenting with AS display multiple instances of this anatomical feature [16]. Regarding the pediatric demographic aged 0–17, the prevalence is estimated to be even higher, up to 20% in CT scans [17]. The splenic hilum remains the most common location for AS [4, 10, 18]. However, many other variations of location can occur, such as the jejunal wall [19], pancreatic tail [10], stomach wall [20], mesentery [21], pelvic cavity [22], and even thorax [23]. Proper recognition of the AS may cause difficulties, especially in cases of hemorrhage and unusual location. Spleen, in general, plays a crucial role as a part of the immune system. However, some hematological diseases, such as idiopathic thrombocytopenic purpura (ITP) and spherocytosis, are indications for splenectomy [24–26]. Thus, total excision of the primary spleen with AS becomes significant for the patient’s well-being and elimination or reduce the symptoms of the disease. The literature describes less than 10 cases of blunt traumatic AS rupture [27–35]. This fact prompted the authors to analyze the injuries regarding their causes, clinical course, and possible preoperative diagnosis or even without surgery and treatment.

## Materials and methods

The desk research was conducted using pre-existing data from other researchers. The study was undertaken as a comprehensive description and was monographic, qualitative, and quantitative. We searched PubMed, EBSCO, Medline, Embase, Scopus, LILACS, BMJ, ClinicalKey, Google Scholar, and Web of Science databases for cases portraying blunt traumatic rupture of the accessory spleen. In the article, we included abstracts and full texts written in any language published until April 2024. In total, we have encountered nine suitable cases for the topic published from 1962 to 2022. Descriptive methods were employed to analyze the collected data. The results are summarized in Table 1 concerning case number, gender, age, details of the trauma, characteristics of the accessory spleen injury, treatment, and others such as previous splenectomy or primary spleen involvement in injury or accompanying abdominal injuries. 

**Table 1:** The cases of traumatic rupture of an accessory spleen

No.	Case author, year, and citation	Gender	Age	Details of the trauma	Characteristics of the accessory spleen injury	Treatment	Previous splenectomy/primary spleen involvement in injury/accompanying abdominal injuries
1	Kumar R, 1962 [27]	M	8	A slip in the bathroom and a fall resulting in a trauma to the lower left thoracic region.	An accessory spleen, about 3 × 2 cm, was found near the lower pole of the spleen with a laceration on its surface, which was bleeding profusely. The pedicle of the accessory spleen had partly torn off the main splenic pedicle near the hilum where there was a large hematoma. Free blood and cloths in the peritoneal cavity.	A diagnosis of appendicitis with peritonitis was made and laparotomy was carried out on the day of admission. Double cut laparotomy– appendectomy with removal of the main and accessory spleen.	Main spleen without any lacerations on its surface. To gain complete control of bleeding, the primary spleen was removed.
2	Fink DW, 1972 [28]	F	n/a	Hit against the point of the dresser with trauma to the left upper abdomen.	Rupture of an accessory spleen.	Surgical removal of the ruptured anterior accessory spleen.	The primary spleen was intact.
3	Richmond R, 1992 [29]	M	32	A strike in the left upper abdomen while playing a softball game.	Isolated splenic rupture– nearly avulsed 1.5 cm accessory spleen. Free intraperitoneal hemorrhage.	Midline laparotomy– accessory splenectomy with ligation of supply vessels. More than 2 L of clotted blood was evacuated from the peritoneal cavity.	The main spleen remained intact.
4	Ashlock SJ, 1998 [30]	M	61	The restrained driver involved in a motor vehicle collision.	Laceration of an accessory spleen with perisplenic hematoma.	Conservative management of abdominal injury (no laparotomy). Operative management of Colles’ fracture.	The patient already had had splenectomy before in 1986 due to previous motor vehicle collision. Liver laceration, left adrenal hematoma, right renal laceration and hemoperitoneum on the CT scan.
5	Karam JA, 1998 [31]	M	19	A strike in left abdominal region during hockey game.	Large perisplenic hematoma with a laceration on the inferior aspect of the spleen. Moderate to large hemoperitoneum. 3 small accessory spleen, forth accessory spleen measuring 2.5 × 2 × 1 cm, was discovered in the splenocolic ligament. The capsule of this accessory spleen was ruptured, with underlying intramural hematoma but no active bleeding.	First conservative management, then exploratory laparotomy. The one accessory spleen was removed by ligating the vascular pedicle at its base.	The primary spleen was intact.
6	Habib E, 2001 [32]	M	36	Car-bicycle collision; the cyclist got knocked over by a car.	There was arterial bleeding from the base of 2 cm diameter accessory spleen located on the splenocolic ligament. Rupture of the capsule of the accessory spleen and perisplenic hematoma.	The accessory spleen was resected. No other visceral injuries or detectable accessory spleens were found. More than 2000 ml of blood was evacuated.	The primary spleen was normal.
7	Tartaglia D, 2016 [33]	M	51	An accidental 2 m fall.	A capsular rupture of a larger accessory spleen (9 cm in diameter) with a large hematoma. The second accessory spleen (2 cm in diameter) undamaged.	Initially, conservative treatment - unsuccessful attempts at angioembolization of the residual splenic artery that vascularized the accessory spleens. Then laparotomy, the larger accessory spleen was excised due to damage. 1000 ml of fresh blood evacuated.	33 years earlier, splenectomy was performed due to traumatic splenic rupture; 2 additional spleens were found in the left hypochondrium and was left intact.
8	Chauvet E, 2017 [34]	M	13	A fall from a kick-scooter (no specified information on velocity) with direct impact of the handlebar onto the abdomen, but no immediate abdominal complaint.	Rupture of an accessory spleen below the lower pole of the main spleen with surrounding fluid collection, hemoperitoneum, no active bleeding.	Managed nonoperatively (no laparotomy), further 1 month follow up with abdominal USG showed a residual hematoma on the inferior pole of accessory spleen	The primary spleen was intact.
9	Wilson T, 2022 [35]	M	57	Motorbike crash. The patient reported losing balance on the bike while riding at 20 km/h and falling heavily onto his left side.	Actively bleeding, ruptured, hypertrophied accessory spleen was found.	Exploratory laparotomy. Accessory spleen removal without complication.	Previous motorbike crash over 25 years ago resulting in a traumatic splenectomy.

## Results

8 men and 1 woman were included in the study. The age range of patients was from 8 to 61 years old, in case of 1 patient the age was not given. The mean age was 35 years old. 2 patients were treated non-operatively with the diagnosis of an AS rupture that was concluded based on radiological examination and one of them already had splenectomy years ago due to the motor vehicle collision. 7 patients underwent laparotomy. Throughout the group of patients managed operatively, in only 1 case, where the main spleen was already removed years ago, the pre-operative diagnosis was precisely concluded to be an AS rupture. In another patient who underwent splenectomy due to trauma years ago, it was concluded to be hypertrophic splenosis rupture. Thus, the diagnosis was basically proper in 2 of 7 cases managed operatively. The remaining 5 cases were diagnosed intraoperatively, but the pathology of the primary spleen had been suspected preoperatively in 4 cases. In 1 case, any pathology of the spleen was not suspected; it was instead an accidental finding during appendectomy, and profuse bleeding was an indication of expanding the scope of the surgery by making the extra cut in the abdomen. However, the necessity of surgical intervention was diagnosed correctly in each case. 3 patients out of 9 (constituting one-third of the cases) suffered from a post-traumatic rupture of the AS during a remote time frame (at least several years later) after the traumatic rupture of the primary spleen and its surgical removal. The rupture of an AS occurred because of traffic accidents, falls, and hit with a blunt subject to the abdomen. No instances of patient mortality were reported in any of the cases.

## Discussion

Although an accessory spleen is a frequent anatomical variation occurring in many locations, it usually holds no clinical significance in the context of healthy individuals. However, the presence of AS becomes significant when planned splenectomy is necessary, such as ITP or spherocytosis, Felty’s syndrome, thrombocytopenic purpura (TTP), hemoglobinopathies, and erythrocyte enzyme deficiencies [24–26]. In such cases, the preoperative recognition and subsequent intraoperative identification with localization of AS are crucial [36, 37]. The presence of AS becomes beneficial for patients suffering from traumatic splenic rupture because the AS can replace the function of the primary spleen. Furthermore, the preserved AS undergoes compensatory hypertrophy. Leon et al. described a case of a 45-year-old woman whose primary spleen was removed after a motor vehicle collision 25 years ago. The AS was left intact. Later, the spontaneous rupture of an AS occurred, and she underwent surgical treatment. The pathologic examination revealed enlargement of remaining splenic tissue from 2 cm to 6 cm [38]. The knowledge about the presence of an AS and its location allows for shortening the time of the surgery. During planned laparotomies, it allows to gain the efficient effect of the procedures by complete removal of the splenic tissue. When it comes to post-traumatic splenectomies, it can omit some preserving procedures of the primary spleen.

Rupture does not singularly encompass the full spectrum of post-traumatic injuries necessitating surgical intervention concerning the AS, as blunt trauma can also cause torsion of the vascular pedicle of the AS. Yoshida et al. described a case of a 12-year-old male presenting left-sided abdominal pain after being beaten in the area. CT scan and ultrasound revealed a 4 cm in diameter, oval mass in the upper left abdomen that was resected 25 days after the injury. It was confirmed to be an AS adhered to the omentum and colon, twisted four times around its axis [39].

Torsion of an AS is an indication for surgery to prevent the risk of future re-torsion and possible bleeding. Twisted AS can wander through the abdomen [40] and mimic other organs’ pathologies, such as bleeding ovarian cysts [41]. Also, infarction of an AS due to the torsion of the mesentery can occur [9]. Torsion of an AS compressing the colon can cause bowel obstruction and should be considered as one of the causes of the acute abdomen [42]. This clinical condition is an indication for an exploratory surgery. However, the proper diagnosis is difficult, especially in critical situations when there is not enough time for appropriate diagnostics. Another uncommon condition requiring the removal of an AS is its torsion mimicking its lymphangioma [43]. Hodgkin's lymphoma can also occur in AS[44]. Littoral cell angioma in the AS can present itself as a spleen rupture. Pilz et al. presented a case of a 73-year-old male with an initial diagnosis of a rupture of an AS and main spleen due to slipping and falling on the left flank at night. The patient underwent surgery, and later, a histologic examination revealed a benign vascular tumor. Thus, after further immunohistochemical exams, the diagnosis of littoral cell angioma (LCA) in the primary and accessory intrapancreatic spleen was made [45]. The accessory spleen is mainly located in the abdomen, but a thoracic location is also possible. The finding described by Suraju et al. was incidental, although alerting. Congenital intrathoracic accessory spleen (CIAS) may pose diagnostic difficulties. If malignancy is expected, the patient should be referred for surgery. Also, to reduce the chance of relapse in patients having a splenectomy for hematologic diseases, resection should be taken into consideration. However, differentiating between CIAS, sequestration, and neoplasm seems challenging when CT scan images appear similarly, and a biopsy might be necessary to distinguish the character of the finding [23].

In our study, in a few cases, the preoperative diagnosis was rupture of an AS. Among 7 patients managed operatively, the diagnosis was clear and unequivocal in case described by Tartaglia et al. [33]. In the second case, it was concluded that the source of hemorrhage was rupture of hypertrophic splenosis, which is almost identical to an AS [35]. Noteworthy, in both cases the primary spleen was already removed. In 5 remaining patients undergoing laparotomy in whom AS rupture could be confirmed micro- and macroscopically and who also had a primary spleen, such a diagnosis was not made before surgery, even in only 1 case. In practice, splenosis can be non-distinguishable from AS before or during surgery. The histopathology exam can distinguish between splenosis and AS. However, in 2 cases, due to conservative management, the diagnosis could not be confirmed macroscopically during surgery or microscopically during histopathological exam [30, 34]. Therefore, the preoperative diagnosis of AS rupture in a patient with an intact spleen seem almost impossible in practice. However, the identification of an acute surgical condition requiring laparotomy or non-operative management was performed and correct in each case. Notably, due to the broad period of the cases analyzed (1962–2022), access to all diagnostic procedures, such as CT, was not available in all cases. Thus, the circumstances were not comparable, and the fair extend was impossible.

Noteworthy, some of the accidents causing AS rupture were quite serious (vehicular accidents), whereas others were seemingly mild, such as a slip and a fall in the bathroom and a hit against the dresser. Some authors did not consider traumatic circumstances like this to be a possible reason for the AS rupture. Teixeira and Hardin described a case of 11-year-old girl horseback riding on the day before admission to the hospital with spontaneous AS rupture [25]. Concerning that some patients had their primary spleen removed due to trauma, and no AS was injured at the same time, we conclude that the energy and power of the trauma are not the most significant for the rupture. Given that the energy sufficient to damage the primary spleen does not induce rupture of the AS, then other factors, such as the mobility of the spleen associated with its fixation to the ligament or mesentery, in conjunction with the length of the vascular pedicle may be involved in the damage to the AS. These structures may confer mobility to the spleen, which facilitates the possibility of accessory splenic rupture during abrupt displacement. AS rupture can occur in many circumstances when there is no injury to any other abdominal organs.

## Conclusion

Based on our research and analysis, we conclude that:

1. Post-traumatic rupture of an AS is a highly infrequent condition that should be taken into consideration when the patient is going to be operated on due to abdominal injury.
2. In practice, diagnosing the rupture of an AS is almost impossible when the primary spleen remains intact, even if diagnostic imaging such as ultrasound and CT has been implemented.
3. The remaining accessory spleen or previous diagnosis of AS presence can facilitate making a proper diagnosis of rupture.
4. Factors such as the power or energy of the trauma are not meaningful for the rupture of an AS.
5. The rupture of an AS is not associated with a rupture of the primary spleen.
6. Abdominal trauma can result not only in accessory splenic rupture and torsion of an AS can occur.

## Data Availability

No datasets were generated or analysed during the current study.
